# Breast ductal endoscopy: how many procedures qualify?

**DOI:** 10.1186/1756-0500-2-115

**Published:** 2009-06-30

**Authors:** Flora Zagouri, Theodoros N Sergentanis, Georgia Giannakopoulou, Effrosyni Panopoulou, Dimosthenis Chrysikos, Garifallia Bletsa, John Flessas, George Filippakis, Alexandros Papalabros, Kostas J Bramis, George C Zografos

**Affiliations:** 1 Breast Unit, 1st Department of Propaedeutic Surgery, Hippokratio Hospital, University of Athens; 114, Vas Sofias Ave, Athens 116 27, Greece

## Abstract

**Background:**

Breast ductal endoscopy is a relatively new diagnostic method with ever growing importance in the work-up of patients with bloody nipple discharge. The ability to perform ductal endoscopy is very important and useful for breast fellows. Learning curve in breast ductal endoscopy remains a *terra incognita*, since no systematic studies have addressed this topic. The purpose of this study is to determine the point (number of procedures during training) beyond which ductal endoscopy is successfully performed.

**Findings:**

Ten breast fellows received training in our Breast Unit. For the training process, an *ex vivo *model was adopted. Fellows were trained on 20 surgical specimens derived from modified radical mastectomy for breast cancer. The target of the education program was to acquire proficiency in performing ductoscopy. The achievement of four consecutively successful ductal endoscopies was determined as the point beyond which proficiency had been achieved. The number of procedures needed for the achievement of proficiency as defined above ranged between 9 and 17 procedures. The median value was 13 procedures; i.e. 50% of trainees had achieved proficiency at the 13th procedure or earlier.

**Conclusion:**

These pilot findings point to approximately 13 procedures as a point beyond which ductal endoscopy is successfully performed; studies on a larger number of fellows are nevertheless needed. Further research, focusing on the learning curves of different training models of ductal endoscopy, seems desirable.

## Background

Breast ductal endoscopy is a relatively new diagnostic method which is rapidly gaining ground with the advent of new generation endoscopies. Patients with bloody nipple discharge stand to benefit most from this method, due to its enhanced visualization of previously undetected areas of the breast ductal system [1,2].

Indeed, the ability to perform ductal endoscopy is important and useful for breast fellows. Unfortunately, the learning curve in breast ductal endoscopy remains a *terra incognita*, since no systematic studies have addressed this topic. Various training models have been documented in the literature based on either *ex vivo *or *in vivo *specimens, but to date questions still remain [3]; is it an easy-to-learn procedure? The purpose of this study is to determine the point (number of procedures during training) beyond which ductal endoscopy is successfully performed.

## Materials and methods

In this study, 10 breast fellows received training in our Breast Unit. All breast fellows had a minimum of three years experience in general surgery and in minimally invasive breast procedures.

**Figure 1:**
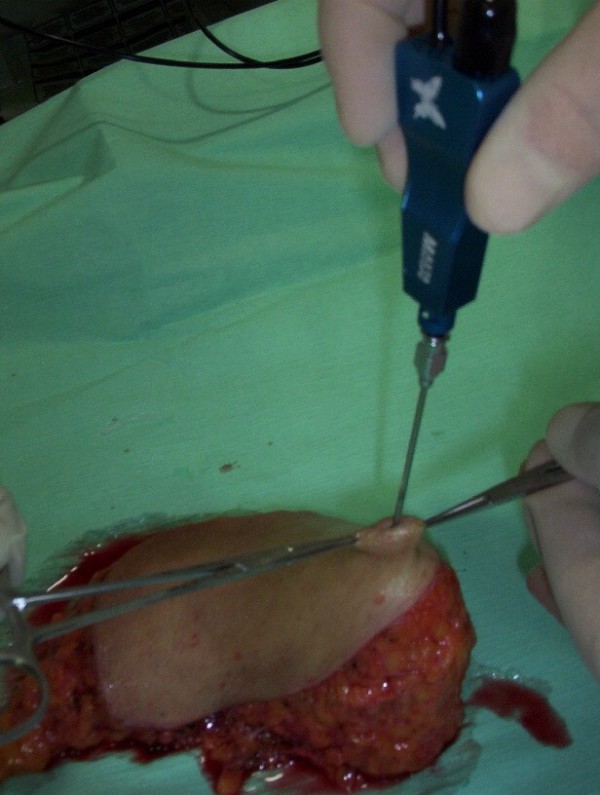
**The specimen is immobilized and the main ducts are aligned**.

For the training process, an *ex vivo *model was adopted. Fellows were trained on 20 surgical specimens derived from modified radical mastectomy for breast cancer. Within 20 minutes of excision, free of any form of fixation and under adequate illumination, the specimens were mounted on a flat surface to simulate the anatomical position on a supine body and submitted fresh to ductoscopy (Mastascope™, Lifeline Biotechnologies, Inc.) (Figure 1).

The target of the education program was to acquire proficiency in performing ductoscopy. The achievement of four consecutively successful ductal endoscopies was determined as the point beyond which proficiency had been achieved.

Ductal endoscopy was considered successful when it encompassed all the following:

i) the ability to perform mapping of the ductal system, recording the location and number of ducts cannulated, taking care to not cannulate the same duct more than once. Additionally, the ability to record the distance of scope advancement and the number of bifurcations passed for each duct.

ii) the ability to locate and identify accessible intraductal lesions.

For each fellow the point of proficiency (i.e. the achievement of four consecutively successful ductoscopies) was noted.

Informed consent was obtained by all participants in the study. Approval was obtained by the local Institutional Review Board.

## Results

**Figure 2:**
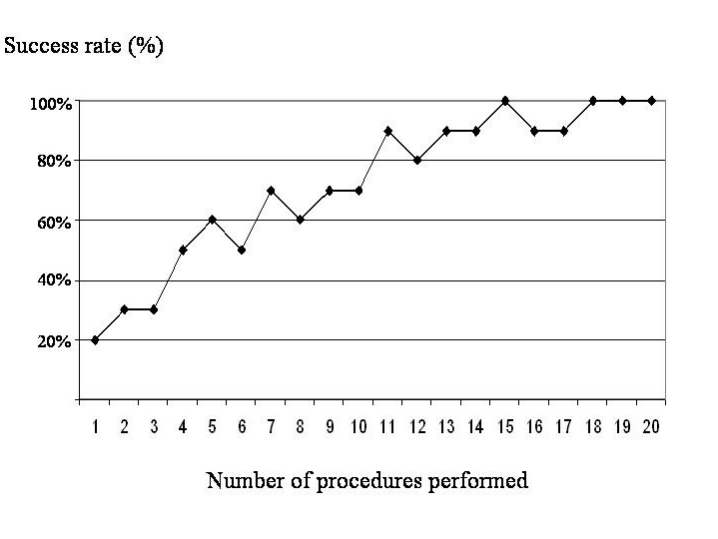
**Success rate of the ten fellows along with the number of procedures performed**.

The number of procedures needed for the achievement of proficiency as defined above ranged between 9 and 17 procedures. The median value was 13 procedures; i.e. 50% of trainees had achieved proficiency at the 13th procedure or earlier. The success rate of the ten fellows at each attempt is depicted in Figure 2.

## Discussion

Ductal endoscopy is a novel method of ever growing importance in the work-up of patients with bloody nipple discharge. Despite its technical feasibility [4,5], ductal endoscopy may be accompanied by significant complications, such as perforation [6]. In addition, women may experience significant discomfort and pain, which may differ along with the strenuousness of the procedure [7]. In this context appropriate *ex vivo *training of the fellows seems indispensable to prevent both complications and discomfort.

Our results indicate that approximately 13 procedures are warranted to guarantee a successful ductal endoscopy. For the optimal interpretation of the result one should bear in mind that ductal endoscopy on *ex vivo *specimens is considerably more complex than on live patients due to less distensible ducts rendering intraductal lesions more difficult to identify. In addition, the adoption of different definitions or settings may diversify the number of procedures needed for qualification.

An important limitation of the study pertains to the definition of success/failure *per se*. The definition integrates a variety of skills (cannulation, passing through bifurcations, ability to locate the intraductal lesions etc); in the present study, '*success*' pointed to the accomplishment of all aspects of the procedure. As a result, no separate rates (success rate of cannulation, number of ducts successfully cannulated, rate of successful passing through bifurcations, inability to reach the needed distance from the nipple orifice, inability to locate the intraductal lesions) have been provided. Indeed, future studies on each component may be particularly interesting, as they may point to the most demanding steps of the procedure.

Notwithstanding, this manuscript highlights the necessity of further studies focusing on the learning curves of different training models of ductal endoscopy. It remains to envisage comparative protocols assessing the efficacy and rapidness of training programs as well as the fellows' perception and attitudes towards the former.

## Conclusion

Breast ductal endoscopy is a relatively new diagnostic method which is rapidly gaining ground with the advent of new generation endoscopies. The ability to perform ductal endoscopy is important and useful for breast fellows. These pilot findings point to approximately 13 procedures as a point beyond which ductal endoscopy is successfully performed; studies on a larger number of fellows are nevertheless needed. Further research, focusing on the learning curves of different training models of ductal endoscopy, seems desirable.

## Competing interests

The authors declare that they have no competing interests.

## Authors' contributions

FZ conceived the idea, designed the study and wrote the manuscript. TNS wrote the manuscript and performed the descriptive statistics. GG, EP, DC, GB, JF and GF participated in the design of the study, made substantial contributions to the acquisition of data and also helped in drafting the manuscript. AP revised the manuscript critically for important intellectual content and made substantial contribution in the interpretation of data. KJB revised the manuscript for important intellectual content and participated in the design of the study. GCZ conceived the idea, designed the study, revised the manuscript for important intellectual content and has given final approval of the version to be published
